# Analysis and prediction of athlete’s anxiety state based on artificial intelligence

**DOI:** 10.7717/peerj-cs.1322

**Published:** 2023-05-25

**Authors:** Lili Guo

**Affiliations:** Physical Education College of Pingdingshan University, Pingdingshan, Henan, China

**Keywords:** RBF neural network, Hierarchical clustering, Competition, Pressure, Anxiety

## Abstract

Obtaining athletes’ anxiety accurately and regulating their psychological state helps improve their competitive performance. Therefore, this article uses a hierarchical clustering algorithm to identify the sources of stress of track and field athletes. A novel and efficient hierarchical clustering algorithm is proposed in this article. The algorithm consists of two stages: dividing and agglomerating. In the dividing stage, the initial data set is taken as a class and subclasses more than the actual number of clusters are obtained through multiple dividing. In the agglomerating phase, the subclasses divided during the dividing process are merged into the correct class. In addition, we construct an analysis model of athletes’ anxiety state based on the radial basis function (RBF) model, where athletes’ anxiety is divided into three categories: physical condition anxiety, competition state and cognitive state. The proposed model is trained from the official website of the China Track and Field Association. The athletes’ information from 500 samples was arranged to form the sample database of athletes’ data. The implicit unit center, function width and connection weight record the characteristics of various sports anxiety states. Then we used the Bayesian and Lagrange models as comparative models for evaluating the psychological state. Precision and efficiency were used for evaluation indexes. The proposed model’s results are much better in accuracy and time than those of the Lagrange and Bayesian models. The outcome of the proposed research can provide a reasonable basis for the decision-making of stress relief for track and field athletes.

## Introduction

Competitive sports are a job with high stress and the failure of high-level athletes in major games often happens. With the professionalization of sports and the improvement of the demands on athletes’ psychological state, it is an inevitable trend to relieve the pressure before the competition. In competitive games, the instinctive reaction of athletes when encountering external stimuli is a non-specific physiological and psychological reaction process. Athletes must relieve high professional stress in sports before the competition. In competitive matches, the automatic response of athletes to external stimuli is a non-specific physiological and psychological reaction process. In track and field competitions, the state of anxiety is caused by pressure, but tension often brings about adverse effects in most cases. Excessive stress will make athletes’ psychological state too sensitive, resulting in slow reactions and seriously affecting the performance of the competition (Zhang & Guo, 2022). During the match, athletes are stimulated by external body sense organs and bear internal psychological pressure, which is more likely to produce tense emotions and anxiety, resulting in excessive tension, inability to coordinate and cooperate between muscles, limb stiffness, body uncoordinated, unable to play an average level, which has a severe impact on competition performance (Neves, Filgueiras Meireles & Berbert de Carvalho, 2017; Timpka, Alonso & Jacobsson, 2014). Different athletes show different psychological pressure (Lopes Dos Santos et al., 2020). If the stress-related emotions in training are defined in a continuous dimension, the stress-related emotions are mainly composed of calm, excitement, boredom and fear. Therefore, it is essential to identify the source of the pressure on athletes in sports competitions.

However, the psychological condition of athletes is usually uncertain and ambiguous, and most psychological services only provide some simple corresponding methods, unable to deal with the cross-complex actual conditions. With the increase in the number of athletes, professionals cannot effectively take into account the psychological condition of everyone. Traditional psychological counseling evaluates athletes’ psychological conditions through consultation by psychological counselors or targeted questionnaires (Boucher et al., 2021; Rice, Olive & Gouttebarge, 2020). However, these methods are inefficient and cannot provide timely and effective counseling advice. Applying the methods related to data mining to psychological pressure coping can solve a large number of athletes with complex and changeable psychological conditions. The rise of artificial intelligence has provided a solution to this problem, with numerous studies using the recognition of physiological signals to assess athletes’ stress levels. Setz et al. (2010) extracted features from the physiological signs of electrodermal response under three different stressors and classified them by DFA and SVM algorithms, with the highest recognition rate of 82.8%.

According to previous studies, track and field athletes may face pressure from various aspects, such as social and personal emotional life (Hollings, Mallett & Hume, 2014). These psychological pressures will make them unable to face the training correctly, depressed mood and very easy to cause sports injuries, so they not only cannot meet the training requirements of the effect but also may ruin their sports career. In addition, most clustering algorithms have the problem of high dependence on parameters and a large amount of computation. This article selected track and field as the research object and proposed an efficient new hierarchical clustering algorithm and combined it with the RBF model to analyze the athletes’ psychological pressure to effectively reflect the impact of athletes’ pressure sources on their competitive level, and provide a favorable basis for the decision making of pressure relief for track and field athletes.

In competitive games, the instinctive reaction of athletes when encountering external stimuli is a non-specific physiological and psychological reaction process. To help psychological experts to provide scientific suggestions, this article carries out automatic simulation from the aspect of data mining, uses clustering algorithm and RBF model to analyze the psychological pressure of athletes, and effectively reflects the influence of pressure sources in the process of competition on the competitive state of athletes, which provides the basis for the decision-making of athletes’ stress relief.

## Related works

### Source of athletes’ stress

Therefore, athletes need to identify the high-stress level in competitive sports. Athletes’ stress can be roughly divided into two categories: sudden psychological pressure or negative influence in competition is called acute stress. But the pressure that already appears in the daily training accumulates unceasingly, called chronic pressure. High-level coaches have gradually mastered the methods to solve the acute stress of athletes, such as keeping encouragement during the competition, giving timely feedback to the referee when the other athletes implement insulting behaviors, controlling the behavior of the audience and reminding the athletes of mistakes and injuries. However, chronic pressure often appears in the field and it is difficult for coaches to correct it through on-the-spot responses. Once many athletes with chronic pressure are exposed to the area, they will lose the game and cause significant damage to the team’s performance. To relieve the regular pressure on athletes, team doctors and psychologists needed to investigate and interview to understand the athletes’ ideas one by one, identify the types of stress through professional psychological analysis, and then deal with them (Myer, Ford & Di Stasi, 2015). In addition, Zhao & Liu (2021) divided the stressors into trait and situational stress according to different types. The former is to deal with the pressure according to the individual’s inertia coping style; the latter is to deal with the needs of individuals combined with their characteristics and environmental factors. Unlike other competitive events, track and field feature short distance and fierce competition, which has higher requirements on Athletes’ physical fitness and speed. In the process of competition, athletes are required to concentrate for a short period, and the improvement of their training results is relatively slow. Therefore, it is necessary to classify and identify the stress sources in the training process.

### Identification method of athletes’ stress

The most effective method to work hard in dissecting the wellspring of stress is fundamental for competitors to conquer mental strain. Considering the low precision of the conventional poll stressor investigation technique, applying a clustering algorithm to stressor analysis can effectively analyze athletes’ stressors (Wu, 2021). However, this method is sensitive to the initial class center selection, which is not conducive to clustering extensive sample data.

The clustering algorithm aims to divide data into a certain number of clusters according to a certain similarity measurement standard. Samples in the same class should have the most significant similarity, while samples in different classes have little or no similarity (Wohwe Sambo, Yenke & Förster, 2019). Currently, there is no unified standard for evaluating clustering algorithm results. Most researchers evaluate the effect of clustering by considering internal similarity and external separateness. That is, data samples in the same cluster should be as similar as possible, while data samples in different clusters should have as little similarity as possible. Both similarities and differences should be examined in a clear and meaningful way. RBF neural network model can effectively solve the above problems. Still, in track and field, the application of this model mostly focuses on the early warning of sports injury risk and ignores the psychological stress of athletes. However, from the perspective of athletes’ stress source identification, the decision chain of the hierarchical clustering method is still insufficient to process relevant data (Li, 2017; Tang, 2009). The RBF neural network model can effectively solve the above problems. Still, in track and field, the application of this model primarily focuses on the early warning of sports injury risk and ignores the psychological stress of athletes.

In addition, many scholars have realized emotion state recognition based on multiple physiological signals by computer. At the same time, when various physiological signals are used for emotion recognition, more features are extracted as far as possible. The classification based on this feature selection will have a better recognition effect. Katsis et al. (2008) designed a wearable emotion recognition system, which provides a method and a wearable detection system to evaluate the emotional state of a racing driver. According to facial electromyography (EMG), respiratory rate (RR), skin electrical activity (PEC) and electrocardiography (ECG), the classification method used in the system is SVM, and the recognition rate is 79.3%. Zhai & Barreto (2006) used the SVM algorithm to extract features of four physiological signals, where two states of “pressure” and “no pressure” are classified, and the highest recognition rate is 90.1%. However, these studies only focus on athletes’ physical characteristics, and cannot analyze other sources of stress, such as competition state and cognitive state, and cannot provide multi-dimensional guidance for athletes’ pre-competition training.

## Analysis of the source of the psychological pressure on track and field athletes

This article mainly proposes a more efficient hierarchical clustering algorithm based on the algorithm framework of CLUBS+ (Mazzeo, Masciari & Zaniolo, 2017). The algorithm in this chapter consists of two parts: the dividing and agglomerating stages. The dividing stage takes the initial data set as a class; through multiple splits, subclasses are obtained more than the actual number of clusters. During the merge phase, the subclasses divided during the split process are merged into the correct class. At the dividing stage, the CLUBS+ algorithm needs to traverse all attribute values of each dimension of the sample in the class and determine the appropriate splitting position according to the changes of relevant indicators. In this article, the algorithm determines the appropriate splitting position through the statistics of the sample distribution of each dimension.

### Problem description

Given a dataset $D = \left\{ {{P_1}, \ldots ,{P_n}} \right\}$, containing $n$ samples, where each sample ${P_i}$ is a $d$-dimensional vector, that is, each sample is composed of $d$ attribute values, and the $i$-th sample is represented as ${P_i} = \left( {{p_l}, \ldots ,{p_d}} \right)$.

The goal of the clustering problem is to obtain a set of classifications $C = \left\{ {{C_1}, \ldots ,{C_k}} \right\}\left( {{C_1} \cup {C_2}, \ldots , \cup \;{C_k} = D} \right.$, and $\left. {{C_i} \cap {C_j} = \emptyset ,i \ne j} \right)$, which makes the sample similarity in the same class as large as possible and in different classes as small as possible.

### Dividing strategy

The whole data set is first taken as a class in the dividing stage. According to the dividing strategy based on sample distribution, the appropriate data set dividing position is found, the class is divided into two parts, and the above dividing process is carried out iteratively until the end conditions of the dividing stage are met. The goal of the dividing stage is to split the whole data set into subclasses that are as similar as possible. The samples belonging to the same class after the dividing stage may be divided into different subclasses, which will be merged into the correct class in the subsequent merging stage. By analyzing the distribution of each dimension of the data set, the dividing dimension of the sufficient algorithm condition and the dividing value in this dimension can be found. Suppose that the dimension where the split position is located in the i-th dimension, and the split value in this dimension is the group where the attribute value h is (h is the median value of the group), then the split position is expressed as (i, h). The two subclasses obtained after the class is divided into two at this dividing position need to meet the following requirements: The maximum number of samples in each one-dimensional sample group of the subclass is greater than the mean number of samples in the sample group of this dimension before the division. The dividing process can be expressed as:

(1) $${C_1} = \left\{ {{P_j}{\rm \mid }{P_j} \in D \wedge p_i^j \le h,i = 1 \sim d,j = 1 \sim n} \right\}$$

(2) $${C_2} = \left\{ {{P_j}{\rm \mid }{P_j} \in D \wedge p_i^j > h,i = 1 \sim d,j = 1 \sim n} \right\}$$

The above process means that data set D is divided into two subclasses D1 and D2 at the split position of (i, h). The dividing process is used iteratively until no split location satisfies the condition is present.

### Agglomerating strategy

After the dividing stage, the initial data set is divided into more subclasses than the final correct number of classes. The task of the merge step is to merge subclasses that belong to the same class into the correct class. The algorithm in this chapter considers that only subclasses of a certain level that are split by the same class need to be considered to be merged, rather than the merging of any two subclasses as the CLUBS+ algorithm does.

**Figure 1:**
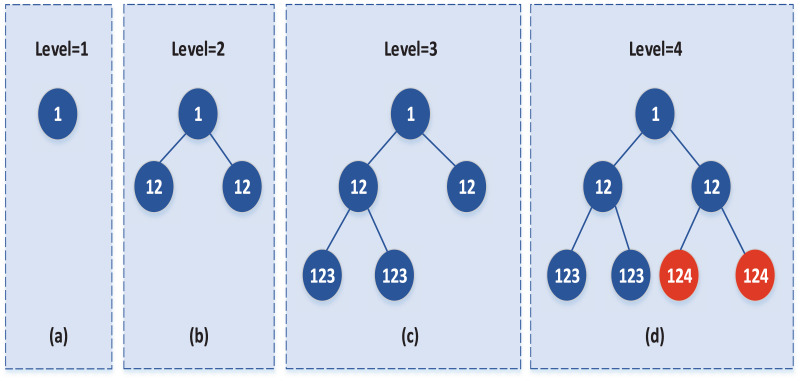
The process of agglomerating strategy.

In the agglomerating process, subclasses are iteratively merged from the bottom up, *i.e*., the merge is detected starting with the last subclass split, then the penultimate subclass, and so on. Until the merge detection of the first subclass is complete. The merge starts with the current number mark and ends with “0”. In the agglomerating detection of each layer, it is not necessary to calculate whether there is a ratio merge between all subclasses on this layer. It is only required to detect that the last r bit (r is equal to the number of bits of the hierarchical mark) in the tag of all subclasses is similar to the subclass of the current level mark. Among these subclasses, the subclass with the most significant mark bit is the one that the current layer should detect and merge. And these subclasses are divided by the same data set at that layer. Therefore, in the merge of each layer, it is only necessary to check whether the two subclasses meet the merge conditions, but not whether any two data sets need to be merged. In addition, if some subclasses are not divided by the same class at some levels, then combining these subclasses at these levels can be done in parallel, thus significantly reducing the amount of computation. The specific merging process can be represented by the binary tree, as shown in Fig. 1:

Each node in Fig. 1 represents a class, and the child nodes of the current node represent the two subclasses of the split node. The number in each node represents the tag of the class. For example, Fig. 1A illustrates the initial data set labeled 1, with the level tag level = 1. Figure 1B represents the two subclasses obtained by splitting the initial data set, and the level = 2 is added after the subclass is marked; that is, the subclass after splitting is observed as “12”, and so on. Figures 1C and 1D and so on. During the merge, the merge detection starts from the current level = 4, and the bit of level at this time is 1 (r = 1), and the subclass whose last 1 bit (r = 1) is equal to four needs to be merged is detected, that is, the red node in Fig. 1D needs to be merged.

**Figure 2:**
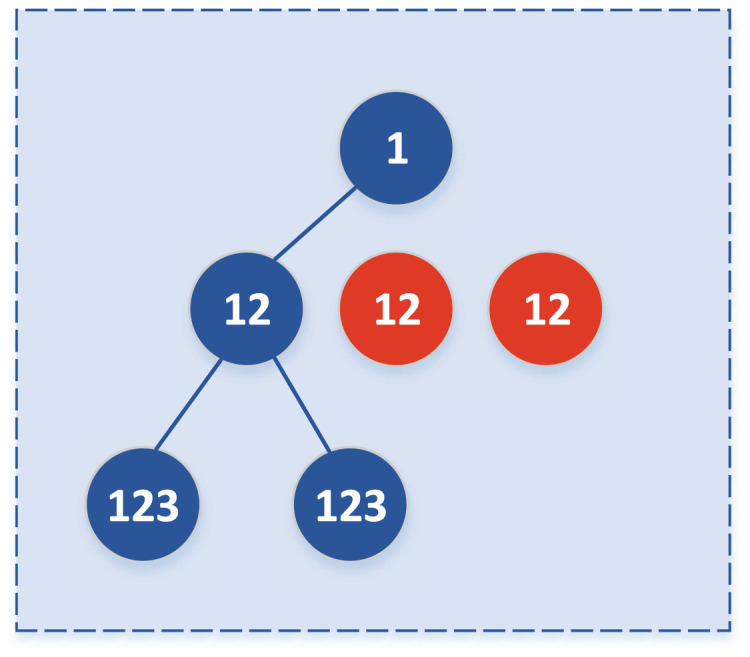
Updated subclasses when they are not merged.

If the two subclasses meet the merge condition, merge the two subclasses and update the merged class tag and level minus 1, level = 3, as shown in Fig. 2C. while if the two subclasses do not meet the merge conditions, then the subclasses will not merge; that is, two child nodes replace the parent node and update the subclass marker and level minus 1. In the subsequent merge, only whether the subclass needs to merge with other data sets can be considered, as shown in Fig. 2:

With the psychological conditions of athletes as the basic sample and the psychological pressure score as the output value, the above model is used to analyze the psychological pressure of athletes (Huang, 2022) to identify the clustering results of various kinds of stress. The identification formula is as follows:

(3) $$Q = {{\left( {\sum\nolimits_{i = 1}^m {\mkern 1mu}  {w_i}{e_i}} \right)} \over {\sum\nolimits_{i = 1}^m {\mkern 1mu}  {w_i}}}$$

## Analysis model of anxiety state of track and field athletes

### Classification of anxiety state

According to the classification of anxiety state of track and field athletes by AHP, the causes of athletes’ anxiety are divided into three types: A, physical state anxiety factor; B, competition state anxiety factor; and C, cognitive state anxiety factor. The specific indicators are shown in Table 1.

**Table 1:** Classification of anxiety state of track and field athletes.

A physical condition anxiety	B competition state anxiety	C cognitive state anxiety
A1 body balance	B1 technical error	C1 opponent strength
A2 injury history	B2 track sound comfort	C2 self-regulation ability
A3 muscle strength	B3 site humidity	C3 match score
A4 exercise load	B4 competition scale	C4 audience atmosphere

This article mainly uses the RBF neural network to distinguish the psychological pressure of track and field athletes according to the input information, dividing them into three categories according to their psychological risk, as shown in Table 2.

**Table 2:** Types of anxiety of track and field athletes.

Output value of RBF	Risk level of anxiety
1	Low
2	Medium
3	High

A classifier is constructed by the RBF neural network, which includes the width of the central layer, the design of the primary radial basis function, the width of the hidden basis function and the weight of the primary basis function. The center and width of the hidden layer of the neural network and the weight between the output layer are determined by the gradient descent method, where the Gaussian function is selected as the activation function of the hidden layer element:

### Hidden layer

In this model, the category of training mode is the anxiety type of track and field athletes so that the hidden layer can be determined, and each category corresponds to a Gaussian function, k = 3. The mean distance between the three kinds of samples and their center is taken as the width parameter of the Gaussian function, and the mean value of the three types of samples is used as the center of each unit.

### Update of weights

The center of the radial basis function and other parameters go through the learning process. Generally, an error correction learning process is adopted, and the gradient descent method is applied. The detailed steps are as follows:

Assuming that There are N sample inputs. For all input samples, define the error function:

(4) $$\xi = {1 \over 2}\sum\limits_{q = 1}^N e_q^2$$

where ${{\rm e}_{\rm q}}$ is the error and is defined as follows.

(5) $$\eqalign{ & {e_q} = {d_q} - y\left( {{x_q}} \right) \cr  & \quad = {d_q} - \sum\limits_{j = 1}^3 {w_{1j}}{R_j}\left( {{x_q}} \right) \cr  & \quad = {d_q} - \sum\limits_{j = 1}^3 {w_{1j}}{{\rm{e}}^{ - {{\parallel {x_q} - {c_j}{\parallel ^2}} \over {2\pi _j^2}}}} \cr}$$

${{\rm d}_{\rm q}}$ is the value of the type required for the sample ${{\rm x}_{\rm q}}$. For example, ${{\rm d}_{\rm q}} = 3$ corresponds to the sample of physical condition anxiety in this article.

### Iteration of free parameters

The iteration process of each free parameter is as follows:

First, output the unit’s weight, as shown in Formula (6) and Formula (7).

(6) $${{\partial \xi (n)} \over {\partial {w_{1j}}(n)}} = - \sum\limits_{q = 1}^n {e_q}(n){R_j}\left( {{x_q}} \right) = - \sum\limits_{q = 1}^n {e_q}{{\rm{e}}^{ - {{\parallel {x_q} - {c_j}\parallel 2} \over {2\sigma _j^2}}}}$$

(7) $${w_{1j}}\left( {n + 1} \right) = {w_{1j}}\left( n \right) - {\eta _1}\displaystyle{{\partial \xi \left( n \right)} \over {\partial {w_{1j}}}}$$

where *n* represents the current variable value, and n+1 represents the value after iteration modification.

Then, determine the center of hidden units:

(8) $${{\partial \xi (n)} \over {\partial {c_j}(n)}} =  - \sum\limits_{q = 1}^n {{e_q}} (n){R_j}\left( {{x_q}} \right) =  - \sum\limits_{q = 1}^n {{e_q}} {{{w_{1j}}(n)} \over {\sigma _j^2}}{\rm{e}} - {{\parallel x_{q - {c_j}(n){\parallel ^2}}^{2\sigma _j^2}} \over {}}\left( {{x_q} - {c_j}(n)} \right)$$

(9) $${c_j}\left( {n + 1} \right) = {c_j}\left( n \right) - {\eta _2}\displaystyle{{\partial \eta \left( n \right)} \over {\partial {c_j}}}.$$

Finally, the width of the function is determined

(10) $${{\partial \xi (n)} \over {\partial {\sigma _j}(n)}} = - \sum\limits_{q = 1}^n {e_q}(n){{{w_{1j}}(n)} \over {\sigma _j^3(n)}}\parallel {x_q} - {c_j}{\parallel ^2}{R_j}\left( {{x_q}} \right)$$

(11) $${\sigma _j}\left( {n + 1} \right) = {\sigma _j}\left( n \right) - {\eta _3}\displaystyle{{\partial \xi \left( n \right)} \over {\partial {\sigma _j}\left( n \right)}}$$

where ${{\rm \eta }_1},{{\rm \eta }_2},{{\rm \eta }_3}$ are learning efficiency, which can be either constant or variable.

### Algorithm flow

The specific process of the learning algorithm is as follows:

Step 1. The number of hidden layer nodes is determined. The average value of all input vectors of the category is taken as the initial center value ${{\rm c}_{\rm j}}\left( 1 \right)$ of the class and the average distance between all input vectors of each category and the center is taken as the initial width value ${{\rm \sigma }_{\rm j}}\left( 1 \right)$ of the class. Each category is input with a sample to solve the initial ${{\rm w}_{1{\rm j}}}\left( 1 \right)$ and set the allowable error ε.

Step 2. Input t training sample to obtain the actual network output ${\rm y}\left( {\rm t} \right)$.

Step 3. Calculate the error between the actual and expected output ${\rm \xi }\left( {\rm t} \right)$. If ${\rm \xi }\left( {\rm t} \right) < {\rm \varepsilon }$, skip to step 6; otherwise, go to the next step.

Step 4. Calculate Formula (6) to equation Formula (11).

Step 5. Update network parameters ${{\rm w}_{1{\rm j}}}\left( {{\rm t} + 1} \right),{{\rm c}_{\rm j}}\left( {{\rm t} + 1} \right),{{\rm \sigma }_{\rm j}}\left( {{\rm t} + 1} \right)$, ${\rm t} = {\rm t} + 1$; if ${\rm t} > {\rm Max\,T}$, no convergence is displayed, go to step 6; otherwise, return to step 2.

Step 6. Finish the whole learning process and save the current network parameters.

## Model training

### Training data

This model is trained from the official website of the China Track and Field Association (athletics.org.cn/main.html). The athletes’ information from 500 samples was arranged, including age, events and awards, *etc*., to form the sample database of pressure analysis of track and field athletes.

### Training process

**Figure 3:**
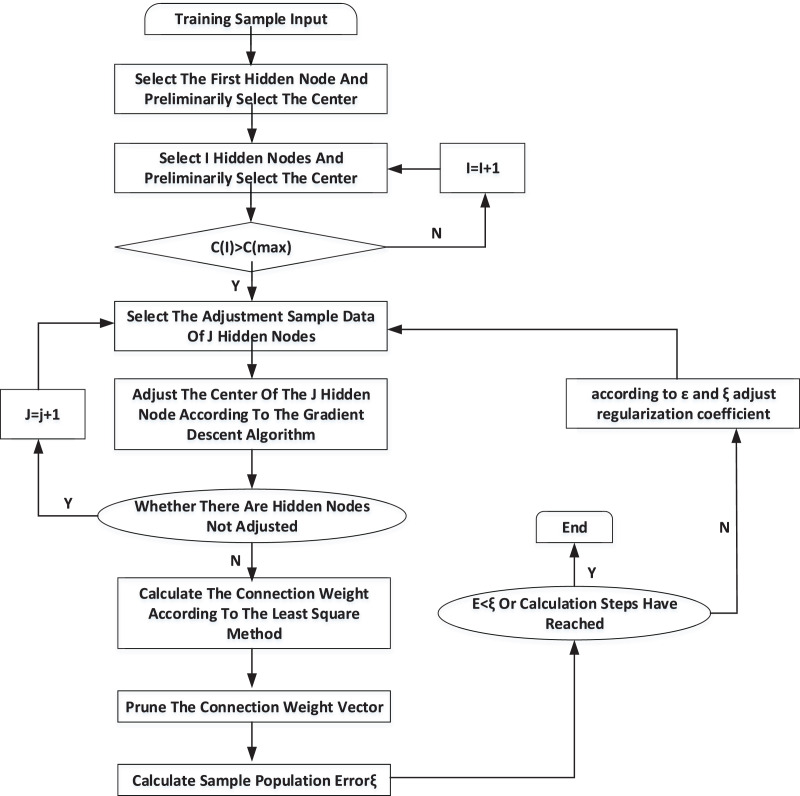
Training process of RBF neural network.

According to the training algorithm and the extracted athletes’ psychological pressure sample database, RBF neural network can start training. Its training process is shown in Fig. 3.

When the neural network is trained, each hidden node’s center, width and corresponding connection weights will not be changed. The related training weights will be stored in the configuration file, and the RBF neural network can enter the working state. The width of the training center and the weights of the hidden neural network and the neural network are recorded. The corresponding value will be output when the data transferred corresponds to a particular risk level in memory.

### Results and Discussion

#### Model training results

**Figure 4:**
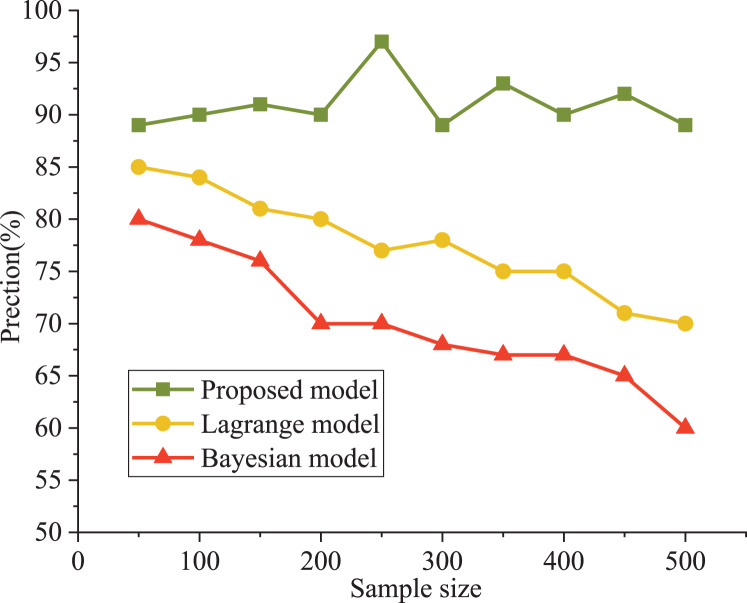
Comparison of model accuracy.

**Figure 5:**
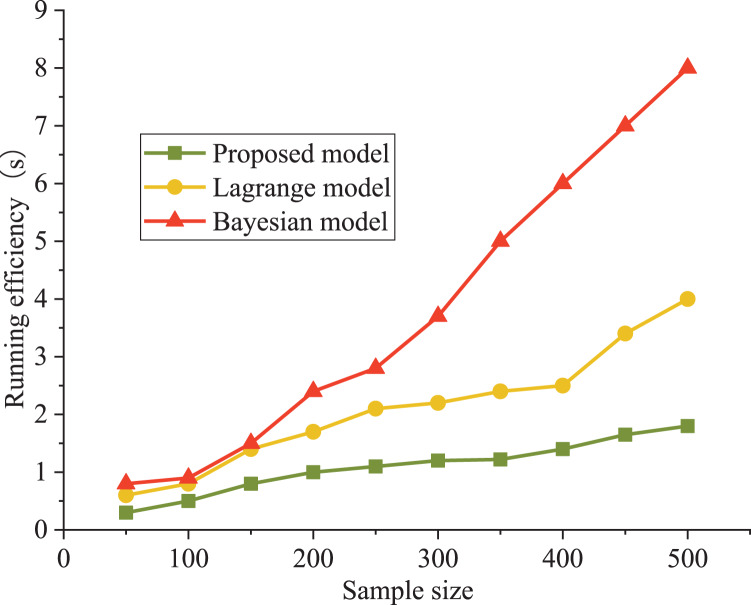
Comparison of model evaluation efficiency.

The Bayesian and Lagrange models are used as comparative models for evaluating an athlete’s psychological state. The precision and running efficiency are selected as the evaluation indexes. The results are shown in Figs. 4 and 5.

It can be seen that the precision curve of the model in this article is always higher than that of the Lagrange model and Bayesian model, which is close to 90%. The accuracy decreased from 81% to 62%. From the objective data analysis, our model’s accuracy varied between 89% and 93%. In addition, Lagrange model and Bayesian model show poor performance under a higher sample size; moreover, the training model’s running time was significantly lower than that of the other two models, with a maximum increase of 37%. The Bayes model has poor performance in running time. When the number of samples is less than 500, the running time is less than 8.3 s, which is about 500% higher than our model (1.4 s).

**Figure 6:**
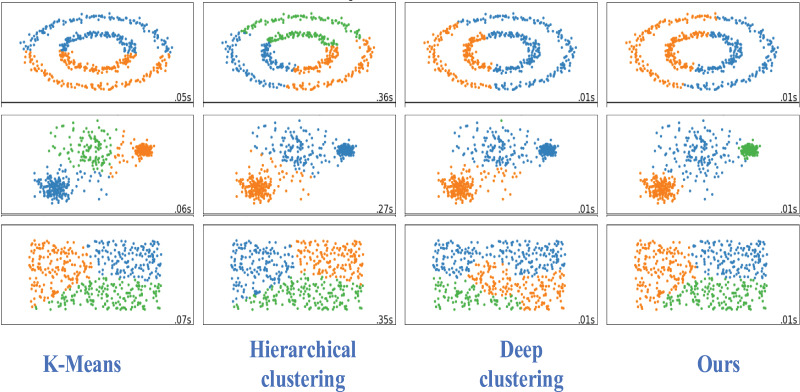
Visualization of classification results.

We visually analyzed the different data sets of the other three methods used for comparison, as shown in Fig. 6.

Our clustering method is superior to the traditional and deep clustering algorithms. Our proposed clustering method has a better clustering effect, more obvious embedding, less overlap, and a better clustering effect for each group of nodes.

#### Classification results of anxiety state

**Figure 7:**
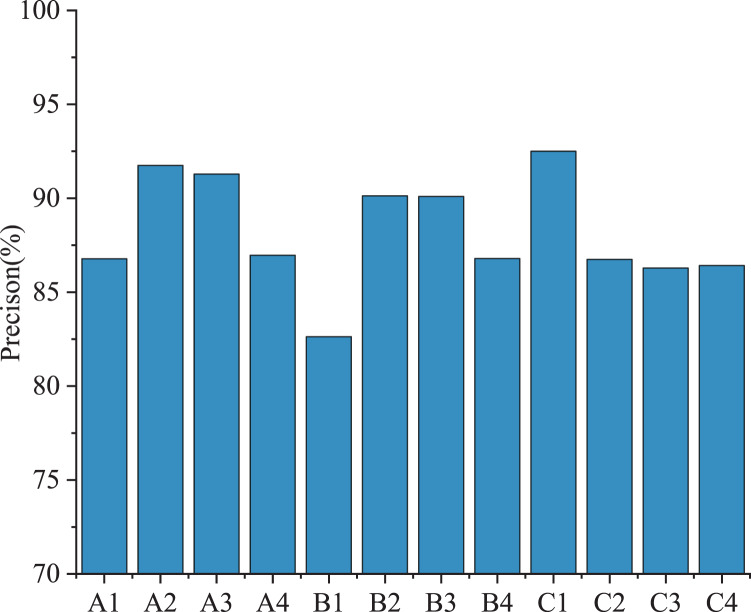
Recognition accuracy of different anxiety states.

Figure 7 shows the recognition accuracy of different anxiety states.

It can be seen from Fig. 7 that the proposed model has a good recognition effect on the pressure source of athletes, and the accuracy rate is higher than 80% in different states. Among them, the fluctuation of physical condition anxiety is more significant, the body balance ability accuracy rate is the lowest (86.7%), and the accuracy rate of injury history is the highest (92%).In the state anxiety of competition, the recognition rate of technical errors was only 82.6%, which may be due to the athletes in the process of competition being too modest and attributing fault to their own mistakes; The accuracy rate of the opponent’s strength in cognitive state anxiety is higher. Similarly, athletes tend to overestimate the opponent’s power due to their modest mentality.

#### Results of psychological stress analysis

Ten athletes of different ages were randomly selected for psychological stress analysis. The original test data were standardized as the score data of [0,10]. According to the identification model of athletes’ psychological stress, the psychological stress data of athletes in the club were obtained, as shown in Table 3.

**Table 3:** Psychological pressure scores of athletes under different factors.

Athletes	A1	B1	C1	A2	B2	C2	A3	B3	C3	A4	B4	C4
M1	7.44	4.56	4.48	7.98	3.45	7.89	8.29	4.55	3.72	7.31	5.61	8.56
M2	3.15	7.38	7.26	5.01	6.38	5.06	3.16	8.26	8.08	4.61	5.46	7.38
M3	4.61	3.80	3.86	8.93	3.60	8.43	6.63	5.90	6.10	6.53	6.53	3.80
M4	7.05	4.55	8.78	4.44	4.43	6.61	7.84	7.34	5.88	4.92	8.81	8.55
M5	3.19	4.94	3.15	6.27	7.07	5.65	3.13	8.98	8.36	6.53	7.68	4.94
M6	8.42	8.29	4.55	3.72	7.31	5.61	4.56	4.48	7.98	3.45	7.89	8.29
M7	8.05	3.16	8.26	8.08	4.61	5.46	7.38	7.26	5.01	6.38	5.06	8.16
M8	8.68	6.63	5.90	6.10	6.53	6.53	3.80	3.86	8.93	3.60	8.43	6.63
M9	8.77	7.84	7.34	5.88	4.92	8.81	4.55	8.78	4.44	4.43	6.61	7.84
M1	7.87	3.13	8.98	8.36	6.53	7.68	4.94	3.15	6.27	7.07	5.65	8.13
Average value	6.72	5.42	6.25	6.47	5.48	6.77	5.42	6.25	7.47	5.48	6.77	8.42

Based on the test results, 0.90 should be taken as the cut threshold of the hierarchical clustering graph. From this, A1, A2, A3, A4 can be combined into one cluster, B1, B2, B3, B4 can be combined into one cluster, C1, C2, C3, C4 can be formed into one cluster. It is proved that the RBF model proposed in this article effectively classifies the anxiety state of track and field athletes. The results of the anxiety analysis of athletes are shown in Table 4.

**Table 4:** Analysis of athletes’ anxiety state.

Athletes	Results of RBF analysis	Results of manual analysis
M1	Competition state anxiety	Competition state anxiety
M2	Competition state anxiety	Competition state anxiety
M3	Physical anxiety	Physical anxiety
M4	Cognitive state anxiety	Cognitive state anxiety
M5	Competition state anxiety	Competition state anxiety
M6	Cognitive state anxiety	Cognitive state anxiety
M7	Physical anxiety	Physical anxiety
M8	Physical anxiety	Physical anxiety
M9	Competition state anxiety	Competition state anxiety
M10	Competition state anxiety	Cognitive state anxiety

From the above data, it can be seen that the classification accuracy of this model on the anxiety state analysis of track and field athletes is high. Except for the M10, the RBF model is consistent with the manual analysis results. Among them, 30% of athletes’ stress in the club comes from physical state anxiety and 30% from cognitive state anxiety, both of which are chronic stress; in addition, 40% of them came from competition state anxiety, which was acute stress. Athletes generally cannot fully concentrate on the game. Most athletes have off-site factors. To help the athletes to deal with the pressure of the competition, the doctors should be focused on the competition.

The conclusion of this article has certain practical significance and academic value. The model can divide athletes’ anxiety into three categories: physical, competition, and cognitive. Then provide psychological counseling countermeasures for professionals. Given cognitive state anxiety, coaches can use relaxation training methods to adjust emotions before the competition, help athletes calm down and make mental preparation before the competition, and can also use relaxation training methods to relieve the athletes’ tension in the process of training and competition, to maintain a stable mental state.

## Conclusion and countermeasures

### Conclusion

This article classifies the sources of athletes’ psychological pressure by the clustering algorithm, and the RBF algorithm is used to analyze the anxiety of track and field athletes. The precision and running efficiency of the designed model are proved to be high by the test sample database. For track and field athletes with different anxiety sources, specific training suggestions can be put forward as follows:

### Suggestions

With the development of sports psychology, it has become an interdisciplinary field combining biology, psychology and digital science. With the importance of sports psychology gradually paid attention to, it is essential to make a detailed, practical and accurate stress assessment for track and field athletes. Therefore, this article classifies the sources of athletes’ psychological pressure by the clustering algorithm, and the RBF algorithm is used to analyze the anxiety of track and field athletes. The precision and running efficiency of the designed model are proved to be high by the test sample database. For track and field athletes with different anxiety sources, specific training suggestions can be put forward as follows:

Given the physical condition of anxiety, the athletes can adapt to the poor comfort experience and modify it to regulate their training behavior to alleviate the discomfort caused by lacking comfort, such as anxiety, fatigue and irritability, and improve the state of anxiety; Given cognitive pressure, coaches can use relaxation training to regulate their emotions before the competition, which can help athletes to calm down and make psychological preparation before the match and can also use relaxation training method to relieve athletes’ tension in the process of training and competition, to maintain a stable psychological state; Finally, because of the anxiety of competition state, coaches can attach importance to the guidance, training and diversion of attention of track and field athletes’ psychological quality, to cultivate their self-confidence, use empathy training methods for the long term, and gradually improve the psychological quality of athletes.

## Supplemental Information

10.7717/peerj-cs.1322/supp-1 Supplemental Information 1 Code and datasets.

## Additional Information and Declarations

### Data Availability

The following information was supplied regarding data availability: The code are available in the Supplemental Files. The data is available at Zenodo: Lili Guo. (2023). Analysis and Prediction of Athlete’s Anxiety State based on Artificial Intelligence (Version V1) [Data set]. Zenodo. https://doi.org/10.5281/zenodo.7533071.
